# A P300-based Brain-Computer Interface with Stimuli on Moving Objects: Four-Session Single-Trial and Triple-Trial Tests with a Game-Like Task Design

**DOI:** 10.1371/journal.pone.0077755

**Published:** 2013-10-31

**Authors:** Ilya P. Ganin, Sergei L. Shishkin, Alexander Y. Kaplan

**Affiliations:** 1 Laboratory for Neurophysiology and Neuro-Computer Interfaces, Faculty of Biology, Lomonosov Moscow State University, Moscow, Russia; 2 Laboratory for Neuroergonomics and Brain-Computer Interfaces, Centre of Converging of Nano-, Bio-, Information, Cognitive and Social Sciences and Technologies (NBICS Centre), National Research Centre “Kurchatov Institute”, Moscow, Russia; ICREA-University of Barcelona, Spain

## Abstract

Brain-computer interfaces (BCIs) are tools for controlling computers and other devices without using muscular activity, employing user-controlled variations in signals recorded from the user’s brain. One of the most efficient noninvasive BCIs is based on the P300 wave of the brain’s response to stimuli and is therefore referred to as the P300 BCI. Many modifications of this BCI have been proposed to further improve the BCI’s characteristics or to better adapt the BCI to various applications. However, in the original P300 BCI and in all of its modifications, the spatial positions of stimuli were fixed relative to each other, which can impose constraints on designing applications controlled by this BCI. We designed and tested a P300 BCI with stimuli presented on objects that were freely moving on a screen at a speed of 5.4°/s. Healthy participants practiced a game-like task with this BCI in either single-trial or triple-trial mode within four sessions. At each step, the participants were required to select one of nine moving objects. The mean online accuracy of BCI-based selection was 81% in the triple-trial mode and 65% in the single-trial mode. A relatively high P300 amplitude was observed in response to targets in most participants. Self-rated interest in the task was high and stable over the four sessions (the medians in the 1st/4th sessions were 79/84% and 76/71% in the groups practicing in the single-trial and triple-trial modes, respectively). We conclude that the movement of stimulus positions relative to each other may not prevent the efficient use of the P300 BCI by people controlling their gaze, e.g., in robotic devices and in video games.

## Introduction

### The P300 BCI and movement

A brain-computer interface (BCI) is a communication system that provides the user with the ability to send messages or commands to the external world without using the brain’s normal output pathways, i.e., without using peripheral nerves and muscles [1]. BCIs are primarily developed as an assistive technology to help people with severe paralysis, but this technology is also increasingly used by healthy people, especially in video games [2]. Within BCI technology, fundamentally new aspects of interaction between the brain and computers emerge because this technology provides completely new “output pathways” for the brain [3]. Operation of these pathways typically requires conscious control, but interestingly, unconscious BCI control is also possible [4].

Currently, the most commonly used BCI is likely the P300-based BCI (the P300 BCI) [5]. In this BCI, available commands are coded by stimuli presented at different locations and times. The user attends the stimuli presented at a location associated with a desired command and ignores the stimuli presented at all other locations, which are associated with different commands. The BCI analyzes the user’s electroencephalogram (EEG), which is typically recorded noninvasively (from the scalp), and can recognize which stimuli are attended because this behavior results in a specific pattern in his or her EEG. As soon as the BCI recognizes one of the stimuli as attended, the system executes the command that corresponds to this stimulus. 

**Figure 1:**
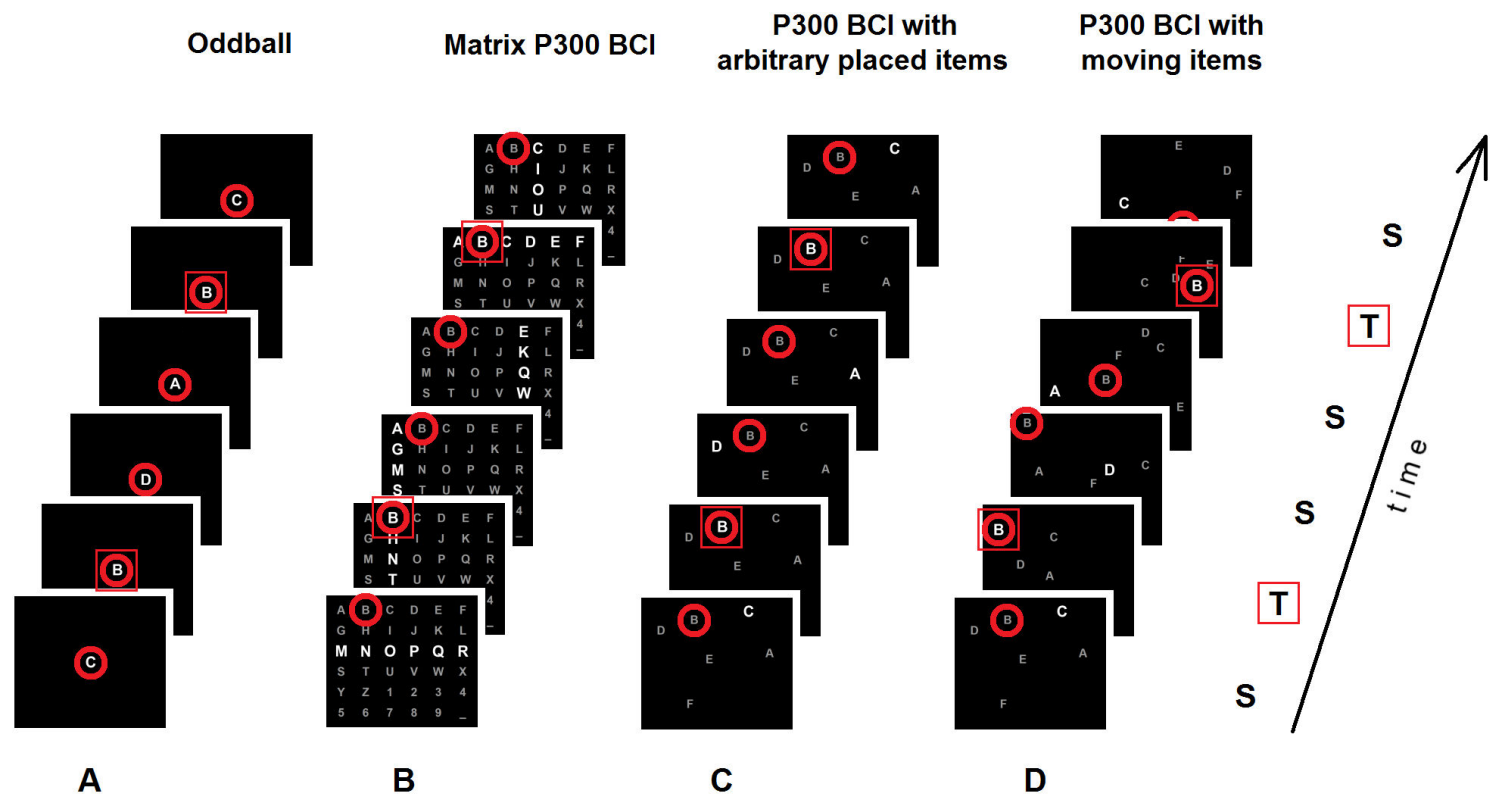
Stimulus spatial organization in the P300 BCI comparing with the oddball paradigm. (A) Visual oddball paradigm. (B) Matrix (“classical”) P300 BCI layout with stimuli grouped into rows and columns. (C) P300 BCI layout with fixed arbitrary stimulus positions and single-cell presentation mode (without grouping). (D) P300 BCI layout with moving stimulus positions and single-cell presentation mode (a design used in this study). S, standards (non-target stimuli). T, targets (target stimuli). In these examples, a flashing letter *B* is the target stimulus. Note that the content at the location that should be attended (marked with a red circle) significantly varies with sequential presentations in the oddball paradigm (A) (both targets and standards are presented there), whereas in the P300 BCI (B, C, D), the attended location can be in one of two states only (target stimulus on/off; the standards are presented at other locations).

All existing variations of the visual P300 BCI design share a common feature: the positions at which stimuli are presented are spatially fixed. The original version of the P300 BCI [6] was developed for spelling, and for this purpose, it was convenient to organize the stimulus positions in a matrix (see Figure 1B below). Most of the current P300 BCIs are also spellers, and it is unsurprising that the matrix design still prevails. Additionally, various new applications of the P300 BCI in which the matrix is used as a “control panel” for entering commands, e.g., for robots or wheelchairs, are common. However, the matrix design is not always appropriate because more freedom is often needed in positioning the locations to be attended for entering commands. Moreover, at least in several applications, *moving stimulus positions* may be useful.

Consider, for example, a user of an assistive or telepresence mobile robot controlled with a P300 BCI. To enter a command, the user must concentrate for a considerable time on stimuli presented on a control panel. After recognition of the command by the BCI, the user’s attention must switch to a remotely located robot to check how the command is executed. The attention then must return to the control panel to enter the next command. These multiple attention shifts not only pose an unnecessary burden on the attentional system (already heavily loaded with the task of attending the stimuli) but also make more dynamic control difficult, e.g., in such situations that require fast canceling of the current operation if an error occurs. Placing the control panel on robotic devices or even placing several of the panel’s elements on separate moving parts of such devices might be a more efficient solution, at least in certain cases. 

Video games are another prospective application to which the standard P300 BCI is not well suited due to this BCI’s static design. At least several of the P300 BCI’s characteristics are certainly suited to gaming applications: the BCI does not require prior training for the user to start operating it, and a very high percentage of people are able to use it [7]. Surprisingly, among the many BCI games already proposed (see, e.g., [2] for a review), only few are based on the P300 BCI technology [8]. The shortcomings of the P300 BCI in its application in gaming are currently being successfully overcome by various means [8]. One of the possible answers to the question of why BCI game developers are reluctant to use the P300 BCI is its static design; games without movement on the screen are relatively rare and often not very engaging.

Stimuli are essential to the P300 BCI. To avoid the division of spatial attention between an important game element and a stimulus, the element and the stimulus should have the same or at least overlapping spatial locations [8]. Further integration of the BCI into a game and further support for maintaining attention to the same location can be ensured if the result of the command entered through the BCI acts upon the elements (e.g., enlarges, transforms, multiplies or destroys the elements) [8]. 

A P300 BCI design that is vivid and flexible compared with standard matrix-based approaches appears to have already been achieved by BCI developers, who proposed presenting stimuli on freely placed virtual objects (e.g., [9-11]) or even highlighting real objects as stimuli [5,11,12]. However, even in these BCIs, the stimulus objects were static, as in all P300 BCI-controlled games described in the literature to date. This feature is a serious drawback of the P300 BCI games compared with games based on other BCIs, such as motor imagery BCI, in which the movement of an attended object is relatively common.

In most popular video games, the visual elements on which attention is focused are typically not static but moving, and that movement plays an important role in making these games engaging. It is difficult to create an attractive game on the basis of the static control panel of the standard P300 BCI. 

However, the P300 BCI can be used without prior training by a very high percentage of people [7], unlike nearly all other BCIs. This feature appears to be particularly important in such a potentially highly marketable application as video games. 

It therefore seems logical to combine the P300 BCI with the free movement of key visual elements in games by attaching the stimuli to these elements. However, such step has not been made to date.

Movement of the P300 BCI stimulus matrix was studied in several cases: in our experiment, targeting the possible influence of movement on event-related potential (ERP) and BCI accuracy [13]; in a P300 BCI game [14]; and in BCI-controlled wheelchairs, in which the matrix position was not fixed relative to the environment in which the wheelchair moved (e.g., [15]). However, in all of these studies, the stimulus positions were fixed relative to each other, which seems to be a serious constraint for game designers and, in certain cases, for designers of BCI control for robotic assistive devices. 

Modifications of the P300 BCI with moving stimuli have been proposed [16-22]. In all of these cases, the initiation of movement and/or the appearance of a moving stimulus were used as stimuli or as a part of a complex stimulus. However, all of these studies described paradigms in which each stimulus moved within a small area, and most importantly, the spatial positions at which the stimuli were presented did not changed significantly from trial to trial. Thus, the basic static spatial design of the P300 BCI was unchanged.

To the best of our knowledge, no journal publications to date have explored the feasibility of a P300 BCI in which the stimuli are presented at positions that move significantly relative to each other.

In the following sections, we will introduce the P300 BCI in more detail and provide arguments showing that existing knowledge was not sufficient to predict whether the P300 BCI would work efficiently when the stimulus positions move relative to each other. Therefore, an experimental study was needed to test the P300 BCI under this condition. We then explain additional goals of our study, i.e., testing the possible effects of multisession practice with such a BCI under other conditions and in single-trial and triple-trial stimulation modes.

### How the P300 BCI works

The P300 BCI was designed by Farwell and Donchin [6] to send commands from the brain to a computer using the P300 wave. This wave, which is also referred to as the P3 wave, is a large positive wave observed in human ERPs approximately 300 ms or longer after the beginning of a stimulus. The wave is elicited when the stimulus is unpredictable or not fully predictable and automatically attracts attention or is voluntarily attended because it requires a certain response, whether overt (motor) or covert (purely mental). In a BCI, such a response can only be covert and has the form of silent counting or just “mental noting” of the stimulus. Later, other ERP components were also shown to be useful in the framework of this BCI [23-29], and several other terms for this BCI paradigm are currently being discussed (e.g., the term “ERP-based BCI” proposed by Treder and Blankertz [29]). 

The standard task used in psychophysiology to elicit the P300 is referred to as the “oddball paradigm”. In this task, different events are sequentially presented to a participant. In the most standard design of the oddball paradigm, several of these events (the targets) are less frequent and require a motor or mental response, whereas the more frequent events (the standards) require no action and can be ignored. 

In a visual oddball paradigm, typical events are visual stimuli, such as images or letters (Figure 1A). A typical visual oddball paradigm with central presentation of stimuli was explored as a part of the P300 BCI design with slow [30] and fast [31] presentation rates. A slightly more specific design was proposed by J. Guan et al. [16], who moved a character string through a window fixated by the user, so that each character appearing in this window could serve as a stimulus. 

However, the “classical” design of a visual oddball, presented in Figure 1A, is not common in the P300 BCI. What is usually referred to as the P300 BCI has a very different look, although this paradigm was developed as a variant of the oddball paradigm. In the original and still most frequently used variant, the user watches a matrix of letters of the alphabet and other characters (Figure 1B). Short-term highlighting (flashes) of rows and columns in this matrix is used as stimuli. The user attends one of the symbols and silently counts (or just mentally “notes”) each time that the symbol flashes as part of either a column or a row flash. Therefore, among the row flashes, one row flash is followed by a large P300 in the user’s EEG, and the same is true for the column flashes. Each stimulus is usually repeated for a reasonable number of times (e.g., 15 in [7]), and the brain’s responses following the stimuli are averaged to ensure a sufficient signal-to-noise ratio. A statistical classifier applied to the averaged signal or to certain features extracted from the signal determines which row and column were attended. The character at their intersection is recognized as the attended character and is spelled. 

Organizing the stimuli in a matrix and the use of stimulus grouping (Figure 1B), already proposed in the first P300 BCI design [6], are efficient solutions for spellers, enabling the fast selection of one command (typing one letter) from many simultaneously available commands (all alphabet in addition to certain other symbols). An interesting recent modification of this original paradigm is referred to as the “checkerboard paradigm”. In this design, the stimuli are organized in a matrix, but rather than flashes of rows and columns, the authors used a specific rule for grouping the flashes [32,33]. An 8x9 matrix is virtually superimposed on a checkerboard (which the participants never actually see), and two 6x6 matrices (white and black) are generated each time from the symbols in the white and black cells, respectively. The stimulus presentation sequences are formed from row and column flashes of the “white” and “black” matrices. To the users, this stimulation appears as random groups of six symbols flashing. Such checkerboard grouping excludes the presentation of symbols of the same group at adjacent positions, leading to a lower number of incorrect selections of symbols adjacent to the target symbol compared with the standard row/column P300 BCI matrix [32]. A generalization of this paradigm, referred to as an “m choose n paradigm”, was previously proposed [34].

As P300 BCIs are increasingly applied to control other computer applications with their own requirements for the interface, such as Internet browsers [35-37] and games [14,38,39] and various devices, including wheelchairs [15,40,41] and robots [42-45], it appears that matrix-like or otherwise spatially structured design and/or stimulus grouping are not always appropriate. “Single-cell” (ungrouped) highlighting can be used for spellers [7,46], but its use appears to be most natural when the number of commands associated with the stimuli is small [9,15,26,47]. However, a single-cell approach can be easily applied for choosing from many commands (including letter typing in spelling) using two or more steps for command selection. For example, in the first step, each cell represents a group of several characters, and the user must choose one such group. In the second step, only the characters from the chosen group are presented, and the user selects one of these characters. Such an approach was used in the “Hex-o-Spell” variant of the P300 BCI paradigm and its modification into designs with more centrally presented stimuli [48] and in the “region-based paradigm” [49]. A specific “lateral” version of the single-cell stimulation design was proposed in [50], in which stimuli are presented alternately on the left and right sides of the screen.

Stimulus positions placed in space without constraints are preferable in certain applications, and particularly in games, even when the stimuli are presented groupwise (several simultaneous stimuli). Single-cell highlighting (stimulation without stimulus grouping) can be used together with matrix design [7,15,26,46], and unconstrained spatial positions can be combined with certain grouping rules [51]. Lastly, single-cell highlighting and unconstrained spatial positions can easily be used together [9–11,38,52] (Figure 1C).

In the BCI literature, the P300 BCI and oddball paradigms are often assumed to be the same from a psychophysiological perspective. However, in contrast to the typical oddball paradigm (Figure 1A), in typical P300 BCI designs (Figure 1B,C), stimuli are presented at different spatial locations. This feature evidently makes the task of differentiation of target and non-target stimuli much simpler. In contrast to the oddball task, there is no need to perceive any details of the events; it is enough to decide whether the events occurred at the attended position. Under the conditions of the P300 BCI task, filtering out the non-target stimuli is facilitated by spatial attention. Users with intact gaze control usually fixate the target, thus having even better perception of events at the target location compared with the non-targets. 

The difference between the perceptual operations required by the two paradigms is likely responsible for the difference between the ERP components preceding the P300 wave. When the standard visual oddball with centrally presented stimuli was directly compared with the standard P300 BCI paradigm with a matrix layout, the P300 did not differ; however, the occipital and occipitotemporal ERP components preceding the P300 wave were very different. In the P300 BCI, but not in the standard visual oddball paradigm, a high-amplitude negative wave was observed at occipital locations in response to targets [27]. The wave is occasionally named according to its latency, such as “N200” (“N” for its peak negativity, and “200” because its latency is approximately 200 ms). Because the latency may vary significantly, the component is also named either N2 or N1 in accordance with ordinal nomenclature. The term “N2”, however, was taken from earlier nomenclature (in which the term N1 was used for the component that is now called C1). We prefer the term N1, consistent with more recent studies (e.g., [53]; [54], p. 37).

This component’s very significant contribution to classification results in the P300 BCI was only recently discovered [25,26,29]. The fact that this contribution was not previously noted by many BCI researchers is likely due to the absence of an N1 subcomponent with good target-non-target discriminative ability at the “classical” locations for the P300 (Pz and Cz). Other issues include relatively little knowledge of the occipital N1 in the psychophysiological literature and an understanding of the P300 BCI paradigm as the equivalent of the oddball paradigm. However, this component was recently found to depend on target foveating, as it disappears if the gaze is not directed to the target [29,55,56]. Therefore, the component is useless under the condition of the most severe paralysis. Nevertheless, the occipital N1 is useful for BCI control by people who can control their gaze. 

### Psychophysiological factors related to stimulus position movement

In the case in which the positions of target and non-target stimuli are moving, their momentary portrait can be the same as in the case of arbitrary stimulus positions (cf. Figure 1 D vs. C). However, in the case of moving positions, the target position will be lost when the stimuli are not present, unless the position is marked. A natural method for such position marking is presenting the stimuli on certain “objects”. The target stimuli are most easily perceived if the moving objects on which the stimuli are presented are fixated and pursued. Pursuit is an important function of the oculomotor system and has been intensively studied [57,58].

Movement of stimulus positions in the P300 BCI does not fully exclude the possibility of using such a BCI by people who cannot control their gaze, which is not rare in the most severe paralysis. A moving target can be tracked without gaze and with attention alone [59], although the efficiency of this tracking is lower than the efficiency of gaze pursuit. 

For the sake of simplicity, in this section and later sections, we will consider BCI use by people with preserved gaze control. Among these individuals, there are many prospective BCI users. In particular, patients with paralysis who are not in a completely locked-in state may be interested in using a BCI to control robotic devices [60,61]. BCI technology may have potential for use as a training tool for several cognitive functions, such as attention, and may thus be useful for even wider groups of people. The widest possible target group is people who might be interested in using a BCI just for gaming.

Surprisingly, the ERP to stimuli presented on moving objects has been studied very little, and only the P300 was analyzed in most cases. One important example is a dual-task target acquisition study [62]. In the experiments, the participants were asked to align a cursor with a target that moved linearly and with constant speed. The target and the cursor were intensified every 1.5 s, each with a 50% probability. In addition to the primary task of cursor alignment, the participants had the secondary task of silently counting intensifications of the cursor or the targets (in different blocks). It was found that the P300 amplitude was much higher for targets than for non-targets in both conditions. However, it is likely that movement could not substantially affect attention to the target in this study because the movement was very slow (it took 30 s to traverse a 20 x 20 cm display positioned 75 cm from the participants). 

In a number of other dual-task studies, the participants were required to perform a tracking task (keeping a cursor centered on a moving target) and to simultaneously count deviant stimuli in an auditory oddball task (see 63 for a review). A typical finding was that the amplitude of the P300 in response to the counted stimuli decreased while the tracking task was being performed compared with the single-task condition (counting stimuli without performing the tracking task). The P300 amplitude did not depend on tracking difficulty. However, in these studies, the P300 in response to visual events was not analyzed.

An exception was a study [64], which used a discrete version of the tracking task and registered the P300 in response to stepwise displacements of the target. When the primary task difficulty was increased, the P300 amplitude in this task (i.e., in response to target displacement) increased, whereas the P300 amplitude in the secondary task (an auditory oddball task) was reduced. From these results, one could infer that the ability of the P300 amplitude to indicate attention to a stimulus can be even improved by stimulus motion. However, as Kok [63] noted, the target displacement eliciting the P300 in the difficult condition in this study was less predictable than in the easy condition, and this factor could be the cause of the effect on the P300 in the primary task. Moreover, tracking a target moving in a stepwise fashion differs from tracking a smoothly moving target. 

In addition, in the target acquisition and target tracking paradigms reviewed above, the target was not only pursued by gaze but also manually tracked. 

Thus, the results of these dual-task studies cannot be used to predict what can happen if motion is introduced into the P300 BCI design.

One could attempt to predict the possible effects of movement on P300 BCI performance using knowledge of the dependence of the occipital N1 and the P300 on cognitive processes and about how these processes are involved or modified when a person pursues a moving target. However, the perception of and attention to moving objects have also been little studied to date [58]. Moreover, different factors may act in different directions when smooth pursuit and/or saccades are used for target pursuit, and the factors’ effects are not simply summated. For example, it is well known that the P300 strongly depends on attention to a stimulus (e.g., [65]). It is also known that attention is deeply involved in pursuit initiation and maintenance and is modulated during pursuit [58,66,67]. Fewer attentional resources can be allocated to attending the stimuli, and therefore, the P300 amplitude might decrease, similar to what was observed in the secondary task in the dual-task studies discussed above (see 63 for a review). 

In our previous work [13], we analyzed a subset of the experimental evidence in the related psychophysiological literature and concluded that even in the case of entire P300 BCI matrix movement (i.e., the positions of both target and non-target stimuli are moving together), the effects cannot be reliably predicted. We therefore performed an experimental study in which the P300 BCI matrix moved in different ways and with different speeds. To the best of our knowledge, this was the only study in which the effects of pursued object movement on the occipital N1 and the P300, responding to stimuli presented on this object, were studied. We found that the amplitude of these ERP components and the classification accuracy did not change in 5°/s and 10°/s movement conditions and slightly decreased in a 20°/s condition compared with a still condition [13]. 

In several studies by other groups, moving and non-moving conditions were not compared, but it was also found that the P300 BCI worked when the stimulus matrix was moving on the screen [14] or was moved against a still visual background, together with the BCI operator, who steered a wheelchair [15,41].

However, separate movement of the objects on which stimuli are presented (Figure 1D) is a more complicated case. The dynamics of convergence and divergence of the non-target objects, expected and actual collisions with the objects and changes in the movement direction of target and non-target objects can impose additional loading on the attentional system. When the moving area is significantly larger than the attended, pursued stimuli, which might be the case in our moving matrix design, attention can be allocated in a substantially different way than in the case of small-object pursuit [67]. Thus, based only on the existing evidence, it is unclear whether the movement of stimulus positions would lead to a strong deterioration in BCI performance. An experimental study is needed to elucidate this question.

### Single-trial BCI games as a possible practice tool

Creating engaging BCI games is one of the possible applications of introducing moving stimulus positions into the framework of the P300 BCI design. A decreased number of stimulus repetitions can also be exploited in P300 BCI games to make the games more engaging [8]. In games, including BCI games, the increased error rate associated with the use of a single trial or a low number of trials could even be beneficial, challenging the player more than conditions guaranteeing high accuracy [68]. 

The need to mediate the translation of intentions into actions by responding to certain stereotyped stimuli that are repeated many times can evidently be disappointing for many potential P300 BCI game players, who may expect a more direct flow of commands from their minds to a game’s virtual world [8]. Similar reasons were assumed to be potentially responsible for the lack of a “presence” sensation in virtual reality due to P300 BCI use [69], in striking contrast to the effects of a motor imagery-based BCI in VR experiments [70]. 

The use of single-trial or low trial-number design might provide an additional benefit. Under such conditions, the user repeatedly receives rapid feedback from the classifier in the form of correct or incorrect recognition of his or her intent. Recent studies showed that “human errors” of various types significantly contribute to P300 BCI classification errors [32,71-77] and that classification accuracy in this BCI depends on psychological factors [78-80]. These findings are unsurprising because the P300 BCI is controlled by the use of attention. Controlled attention and the stability of the related P300 responses may suffer from “attentional lapses” and “mind wandering”, and in turn, these phenomena might be affected by other psychological factors [80,81]. Evidence that ERP amplitude can be controlled using operant conditioning in a single-trial ERP design exists [82,83] (but also see 84). It seems logical to expect that feedback from the classifier might be helpful to improve the signal-to-noise ratio in the EEG through unconscious physiological mechanisms of conditioning, through conscious adjusting of the user’s strategies or through other means. As the P300 wave and several other ERP components are linked to attention, we even may hypothesize that attention improvement within the P300 BCI task or even more general improvement in attention can be achieved after sufficiently long and intensive practice with the P300 BCI. The moments at which attention control is lost are often unnoticed [85]. Thus, instruments for revealing failures in attention control can be considered as candidates for use as attention-training instruments. 

Finke et al [38] proposed to use P300 BCI games to provide rich interaction possibilities, which can be useful to study the possible effects of neurofeedback. In the authors’ BCI game, feedback was enriched not only using a single-trial mode of interaction but also by making the BCI-mediated action dependent on the classifier output. The character controlled by the BCI frequently moved due to the single-trial mode, but the stimulus positions were still. Within a single-session study, the authors observed a degree of improvement in BCI control in the game condition (with feedback) compared with the level observed for data recorded during classifier training without feedback. However, the design of the study did not exclude contributions from factors unrelated to feedback, such as adaptation to the experimental conditions or increased attention in the gaming conditions compared with the less engaging classifier training condition. 

To our knowledge, the single-trial BCI experiments described in the literature to date were always conducted in a single session. The same is true for the attempts to condition the P300 wave amplitude in feedback experiments, which yielded controversial results [83,84]. The long-term use of the P300 BCI by highly motivated patients was reported [86]. However, a patient’s use of the BCI is not a valid test for the hypothesis regarding practice effects beyond simple adaptation to a task, for the following reasons. Patients are offered a relatively high number of trials, which are averaged to decipher each command, keeping the number of errors low. The low number of errors means that negative feedback is only rarely given and that a short-term loss of attention will not lead to an error due to compensation from the other periods of time. If an error occasionally appeared due to insufficient compensation, the user would likely not detect the difference between the “compensated” and the “non-compensated” cases, so even this rare negative feedback might not help to improve attention and/or BCI accuracy. Furthermore, because the mental state and the related brain state may vary between many trials and because the feedback shows only the averaged result, the user cannot be certain in which trial(s) he or she “behaved” incorrectly (e.g., was not sufficiently concentrating), leading to an error. In addition, several factors affect the P300 amplitude and P300 BCI accuracy as a function of preceding target-to-target intervals (for a review see, e.g., 76). All of these factors makes even less informative the feedback about the “efficiency” of various specific mental states, i.e., about states’ ability to produce high-amplitude responses.

The feedback provided by correct/incorrect action in the P300 BCI may help to improve BCI control (and possibly attention control) more efficiently if a single-trial BCI mode is combined with repeated BCI use. However, special efforts should be made under this combination of conditions to avoid a loss of interest and to maintain high attention to stimuli across the series of sessions. These goals can be achieved using a BCI game and making this game sufficiently engaging. Moving stimulus positions could help to address these aims.

### Objectives of this study

In preliminary experiments, we presented flashes on circles (“balls”, 1.2° diameter) that were either moving or still. The P300 and N1 amplitudes did not strongly differ between these two conditions. 

These preliminary observations suggested that a degree of non-random control may be achieved by the P300 BCI under moving conditions. Testing this hypothesis was the first objective of our study, and the hypothesis was confirmed.

The second objective was to ascertain whether healthy users can maintain interest in a task across several sessions when using a game-like P300 BCI with moving stimulus positions in single-trial mode or at least few-trial mode (specifically, triple-trial). In both modes, stable interest in the task over four sessions run in different days was reported by most participants.

In addition, the study was considered as a preliminary test of the possible beneficial effects of the intensive use of single-trial and triple-trial BCIs on BCI classification accuracy and the amplitude of ERP components. We also hypothesized that such effects (if these effects are already revealed after several sessions) would be stronger in the single-trial mode compared with the triple-trial mode (having similar temporal characteristics) because no averaging is used in the former mode. However, none of these effects was observed.

Parts of this work were included in our conference paper [87]. Certain data from these experiments (not specific to the movement of the spatial positions of stimuli) were used in an analysis of ERP amplitude dependence on the temporal position of stimuli in stimulus presentation train [88]. In the present study, much more detailed results are presented, including the results of an ERP analysis.

## Methods

### Participants

In total, 12 unpaid, healthy volunteers participated in the study after signing an informed consent form. The volunteers were randomly assigned to two groups: the ST group (*n* = 6, 4 females), which practiced in *Single-Trial* BCI mode, and the TT group (*n* = 6, 5 females), which practiced in *Triple-Trial* mode. In each group, ages ranged from 19-23 years, and the median was 21. All of the participants had normal or corrected-to-normal vision. The experiments were run in accordance with institutional guidelines and the Declaration of Helsinki. The experimental protocols used in this study were approved by The Bioethics Committee of the Faculty of Biology, Lomonosov Moscow State University.

Each participant completed four sessions scheduled on different days. The participants were informed that they could cancel participation in future sessions at any stage; however, none of these individuals used this option. The minimal interval between two sessions was two days. The median interval between consecutive sessions for the 1-2, 2-3 and 3-4 pairs of sessions was 12.5, 12.5 and 19.0 days, respectively, in the ST group and 11.0, 16.5 and 15.5 days, respectively, in the in TT group.

### Data acquisition

Data acquisition, stimulus presentation, online signal processing and classification were performed by an in-house Python program. EEGs were recorded at a 500 Hz sampling rate at Cz, Pz, PO7, PO8, O1 and O2 against a reference at the right earlobe, with a ground electrode at Fpz. Potentials at the left earlobe and at a position 2 cm above the outer canthus of the left eye (used for electrooculogram (EOG) recording) were recorded along with the EEG with the same settings. The stimulus information and online classifier output values were also saved for each trial. 

Synchronization between the stimulus trigger recorded using the BCI software and the actual stimulus presentation time (see [89] for a discussion of related serious issues in P300 BCI implementations) was estimated by a synchronization test prior to and immediately after each session. Connections between the EEG acquisition device and the computer that presented stimuli and recorded data were uninterrupted throughout each session (including the time for this test). During the test, luminance changed in the upper right corner of the screen in synchrony with target-ball flashing. A photodiode-based sensor registered these changes, and its signal was recorded as one of the EEG channels. The observed timing error for each of 200 flashes recorded before and 200 flashes recorded after each session was always within -12…+4 ms. The test indicated that the synchronization was sufficiently precise for ERP analysis. 

### Stimulus design

The stimulus display was presented on a 17 in CRT monitor (ViewSonic GT775) with an 85 Hz refresh rate and at a distance of approximately 85 cm from the participant’s eyes.

**Figure 2:**
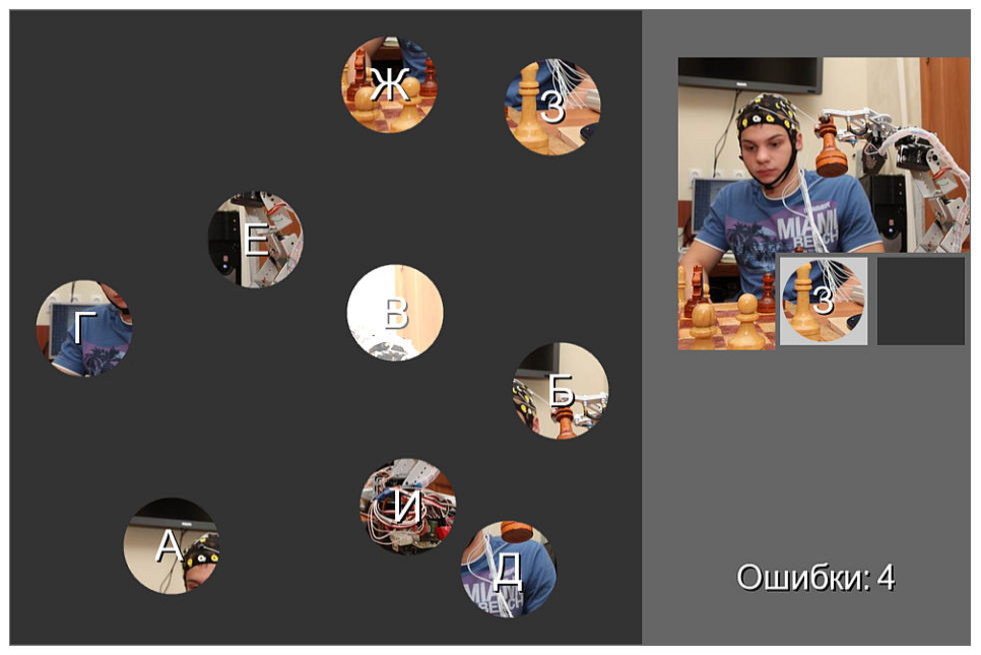
Example of the P300 BCI game *Billiard* *Puzzle* display. Each “bouncing ball” contained a fragment of the picture being assembled on the right. The correct order of targets is cued by letters of the Russian alphabet. The current target is marked by a circle in the right panel. The ball with the letter “B” is flashing here. A counter at the bottom right indicates the number of errors (here, four errors). Note that this display follows the scheme presented in Figure 1 D (moving stimulus positions and single-cell presentation mode).

The main difference between our BCI and the standard P300 BCI is that the positions at which the stimuli are presented can move in our BCI. More specifically, in the implementation used in this work, the stimuli permanently moved throughout the user’s observation of the BCI display on the screen. To facilitate the stable tracking of these positions, the positions were made visible in the form of nine “bouncing balls” (Figure 2).

From the participant’s eye position, each ball was viewed as a circle with a 2.2° diameter moving linearly at a speed of 5.4°/s. The size of the field within which the balls moved was 13.9° x 13.9°. “Collisions” of balls with each other or with the margins of the field changed the balls’ movement direction in a naturalistic way (assuming elastic collision and equal mass of the balls). More specifically, the balls changed their movement direction when they hit each other, and in the case of collision with a margin, the angle of reflection was equal to the angle of incidence. Except for changing direction at the moment of collision, each ball always moved along a straight trajectory. The individual ball movement directions were defined randomly when the balls appeared on the screen, and the individual trajectories were then modified only by the collisions. 

Details regarding what occurred after ball selection by the BCI are given in the next section (“Procedure”).

The balls contained fragments of a picture that was assembled in the right panel on the screen (see Figure 2). Similarly to our previous (static) P300 BCI game, *MindPuzzle* [8,39], the goal of the player in the new game, *Billiard Puzzle*, is to assemble a full picture from these fragments, whose highlighting is used as stimulation. 

The balls were labeled with the first letters of Russian alphabet (all participants were native Russian speakers), indicating the order in which the letters should be selected to fill the cells in the matrix in the assembled picture panel (on the right of Figure 2; for more details on how the target was indicated for each run, see the next section). The labels were used to help the participants to correctly identify current targets.

In each session, 10 different pictures were offered for assembly, with one picture per “game”. Pictures were never repeated for the same participant, even in different sessions. Together with the pictures used for practice, 48 pictures were used for each participant. This pool of 48 pictures was the same for all of the participants, but the order was random for each participant. The pool was formed from attractive, colorful photographs showing animals or, more rarely, fruits, plants or cars from which sufficiently different round fragments could be obtained. 

A stimulus (“flash”) was an increase in the brightness of a ball (see an example at the top of Figure 2) for 125 ms. Balls flashed in a random order, without pauses between the flashes. Each time, only one ball was flashing, so the paradigm was an analog of the single-cell stimulus presentation mode in the static P300 BCI [7,9,26,38,46]. 

The time needed to find the target in the game phase varied significantly, and it was important to provide the participants with a means by which they could start stimulation themselves. In this case, the participants could delay the initiation of the stimulation not only until finding the target but also until feeling that they were “ready”. This feature may be valuable in helping participants to become more sensitive to their mental states in the sense of supporting or not supporting accurate control of the BCI. “Asynchronous” control of the P300 BCI is possible [90,91], although the use of such technologies could make interpretation of the experimental results more complicated. The specific experimental task used in the present study was more of a model of a BCI game for healthy people rather than an assistive tool for paralyzed individuals. In BCI games, combining BCI control with usual control based on physical movements is natural and has already been used in P300 BCI-based games and a game-like virtual reality control scenario [8,47,52]. We therefore decided to provide the participants with a mouse-based initiation of stimulation. Stimuli started 3 s after the mouse click. No mouse, however, was used in the calibration phase, in which the targets were directly indicated. 

According to common terminology in P300 BCI studies, the presentation of all of the stimuli (in our design, on each ball), each flashing once, formed a stimulus “sequence” (1.13 s in length, as the number of stimuli was nine). Within each sequence, the order of flashing balls was random, with the constraint that the same balls did not flash twice in a row, even in multitrial modes. If more than one stimulus sequence was used per run, the stimuli followed each other without pauses. Flashes and ball movement were independent of each other.

### Procedure

Each session consisted of two main phases. In the calibration phase, data were collected for classifier training. In the BCI game phase, the classifier was applied to online detection of the participant’s intent. In addition, the first session started with a short demonstration and practice in counting the target stimuli.

The calibration phase consisted of 15 runs. A target ball was randomly chosen for each run but was never repeated in a row. The ball was highlighted for 2 s by rapid (5/s) flickering. The stimuli started 2 s after this flickering ended. Eight stimulus sequences were used per run. Runs were separated by 10 s pauses, including the time spent on indicating the next target. The total duration of the calibration phase was 4 min, 45 s. During this phase, the participant’s task was to fixate on the letter on the target ball, to count or silently “note” (his or her choice) the ball’s flashes and to pay no attention to the flashing of all other balls. The participant was asked to count or note the flashes in a “clear” and “emotional” way but also to be relaxed.

After the calibration phase ended, the classifier was trained, and a short practice in online mode was run. After a short break, the game phase then started. This phase differed between the ST and the TT groups of participants based on the number of stimulus sequences used per run. The ST group played the BCI games in Single-Trial mode, i.e., with one sequence per run, and the TT group played the games in Triple-Trial mode, i.e., with three consecutive stimulus sequences per run. 

In all of the modes in the game phase, the participants followed the same instructions as in the calibration phase, which were related to following the target ball and counting or noting its flashes. In addition, operating the BCI was organized in the form of a game, with the aim of assembling a full puzzle from the fragments presented on the balls. The participants were encouraged to achieve as high a score as possible while playing these games and to assemble the puzzles in full whenever possible. 

An attempt to assemble one picture was defined as one “game”. Thus, 10 games were played per session. Games were separated by 1-2 min breaks, and an additional 5-10 min break was used between the 5th and 6th games. 

To avoid contamination of the data by the effects of factors related to, e.g., the incorrect identification of the targets needed for puzzle assembly and variations in individual task-solving strategies, the order in which the fragments should be selected was fixed but clearly indicated by lettered labels (the target order was always alphabetical) and by highlighting the current target in the assembly matrix (see Figure 2). The matrix always had to be filled from left to right and from top to bottom, additionally supporting easy identification of the correct target.

After finding the target, starting to pursue the target with gaze and preparing to attend the target stimulus, the participant pressed a mouse button, initiating start of the stimulation in 3 s. During stimulation, a participant’s task was exactly the same as in the calibration phase; however, the stimulation was shorter (1.13 s in Single-Trial mode and 3.38 s in Triple-Trial mode). To reduce spontaneous reactions to other balls’ flashing and artifacts related to saccades, the participants were asked to pursue the target after a single target flash in the Single-Trial mode and after the third target flash in the Triple-Trial mode, until the ball classified by the BCI as a target was highlighted. The delay between the end of the stimulation and feedback (highlighting of the chosen ball) was 2 s, and the duration of highlighting was also 2 s. In the case of a correct choice, i.e., when this highlighted ball was indeed the target, the corresponding cell of the matrix in the right panel on the screen was filled, and the fragment in the cell next to the previous cell became the new target. Otherwise, the error counter showed an increase of one, and the target remained the same as in the previous run. The correctly recognized ball remained inside of the field and continued moving and flashing, similar to other balls. Thus, the total number of moving and flashing balls remained the same and was always equal to nine.

“Winning” the game meant that the full puzzle was assembled, i.e., all nine items were successfully completed one by one. The number of attempts was restricted not for the individual targets but for the game in total, so the game was “lost” if the counter showed 10 errors. These rules constrained the minimum number of selections made in one game to nine and the maximum number to 18. The range of possible numbers of selections per session was between 90 and 180 (except for the 4th session; see the description of the *Test mode*, below). The number of assembled puzzles and the total number of errors were announced to the participant at the end of each session.

The second half of the 4th session (five games, i.e., selections 46 to 90) was run for both the ST and the TT groups in an additional mode designated as the *Test mode*. This mode was also a game and differed from the main Single-trial and Triple-trial modes only by the number of stimulus sequences, which was five in this case. The Test mode was designed to imitate the real-life use of a BCI, in which higher accuracy is needed, and to provide a tool for comparing the results of hypothetical improvement in BCI control skills. We expected that if the practice in the Single-Trial And Triple-Trial modes was not equally efficient, this difference would be reflected in the accuracy demonstrated in this mode. The participants were informed that this mode was an important part of the experiment and that it assessed the results of their previous practice. The participants were also informed that in this mode, they may achieve their best results due to easier recognition of their intentions by the BCI. 

### Classifier

No specific artifact correction or rejection procedure preceded classifier training and online classification because it was found that pursuing similar moving targets did not lead to strong EOG contamination of the data in pilot experiments. 

For both classifier training and online classification, the EEG was filtered in a 1-10 Hz band with an FIR filter and decimated down to 20 Hz, and 1 s epochs starting from the stimulus onset were extracted. The amplitude values concatenated for all six EEG channels formed a feature vector. The number of target and non-target epochs for classifier training was 120 and 960, respectively. Classifier weights were obtained by Fisher discriminant analysis. During the main part of the experiment, the weights were applied to each epoch separately. In the Single-Trial mode, the attended ball was determined by the highest value of the classifier output. The same rule was used in the Triple-Trial mode and the Test mode, with the only difference being that the classifier outputs were first averaged for each ball separately across the three and five trials, respectively.

### Classification accuracy

*Overall classification accuracy* was computed as the percentage of runs in which the ball classified as a target by the BCI matched the target designated for this run. 

This measure, however, could be affected by differences between balls in the probability of producing a well-classified ERP. All of our participants reported that the balls seemed not to be equal in the sense of the subjective easiness with respect to noting the balls’ flashing, and most participants associated several of their failures with “bad” balls. In certain cases, these “bad” balls contained a very bright fragment of a picture. In other cases, the fragment of a picture was too homogenous. Several of these features could possibly lead to deterioration in fixating the target and/or failure to detect its flashing. Failures associated with such “bad” balls could negatively bias overall accuracy because the participants were required to continue their attempts to select the same ball during the next run after each failure. To have an estimate not confounded by such effects, a corrected estimate of accuracy (*corrected classification accuracy*) was computed in the same way as overall accuracy but only for the first attempt to select each target ball. That is, the corrected classification accuracy was the percentage of successful target selection in the runs in which a target was presented for the first time.

Both accuracy indices were computed per participant and session. An exception was made for the 4th session, for which the indices were computed separately for the first half of the session (Single-Trial or Triple-Trial mode) and for the second half of the session (Test mode). 

### Interest

Participants were asked to rate their interest in the task (“generally over today’s experiment”) by putting a mark on a visual analog scale (VAS) at the end of each session. The scale was a horizontal line of 100 mm in length that was anchored by the words “not interesting” at the left end and “extremely interesting” at the right end. The distance between the mark and the left end was measured after the experiment in mm, giving the estimate of the corresponding variable (in arbitrary units). Thus, 0 meant the lowest interest (no interest at all), and 100 meant the highest possible interest. The scales for sessions 1-4 were located on the same sheet of paper, one under another. This setup allowed (and even encouraged) the participants to find a position for the new estimate in relation to the previous estimates.

### ERP analysis

Offline analysis was performed with MATLAB® (MathWorks, USA). The EEG and EOG channels were re-referenced to the average of the two earlobes and bandpass filtered using a 2nd-order Butterworth filter in the forward and backward directions (for zero phase shift) in the range of 0.5-20 Hz, and epochs -0.2...0.6 s relative to the stimuli were extracted. Epochs with an amplitude exceeding ±50 µV in any channel were excluded from the analysis. The percentage of rejected epochs per participant and session never exceeded 11%. Visual screening of epochs extracted from non-filtered EEG confirmed that no significant artifacts escaped this procedure. Notably, strong artifacts from saccades were not common in our data, likely because pursuing the moving balls required only smooth-pursuit eye movement and small saccades. Blinking artifacts were also rare, likely because the stimulation periods were short.

The epochs were averaged separately for target and non-target stimuli per subject and session. For accuracy, in the case of the 4th session, the average values were computed separately for the first and second halves of the session, i.e., for the Single-Trial or Triple-Trial mode and for the Test mode, respectively. 

For the analysis of the N1 component, the PO7, PO8, O1 and O2 channels were averaged together. The N1 peak amplitude was estimated using this averaged signal as the maximum value in a 120-250 ms interval. The amplitude of the P300 peak was estimated as the maximum in the 250-500 ms interval at Pz. No baseline correction was used because filtering removed most of the slow variations in the signal. A slow negativity remained, however, in the beginning of the ERP waveforms. Averaging of longer epochs separately for different target positions within a stimulus sequence showed that this negativity started approximately 0.5 s before the first stimulus in the sequence and tended to grow if the target appeared later in the sequence (i.e., if the target-to-target interval was long). Therefore, the negativity may correspond to expectation of the target stimulus. No measures were taken to remove this component from the signal because the negativity seemed to disappear before the P300 wave reached its maximum.

## Results

### Classification accuracy

**Figure 3:**
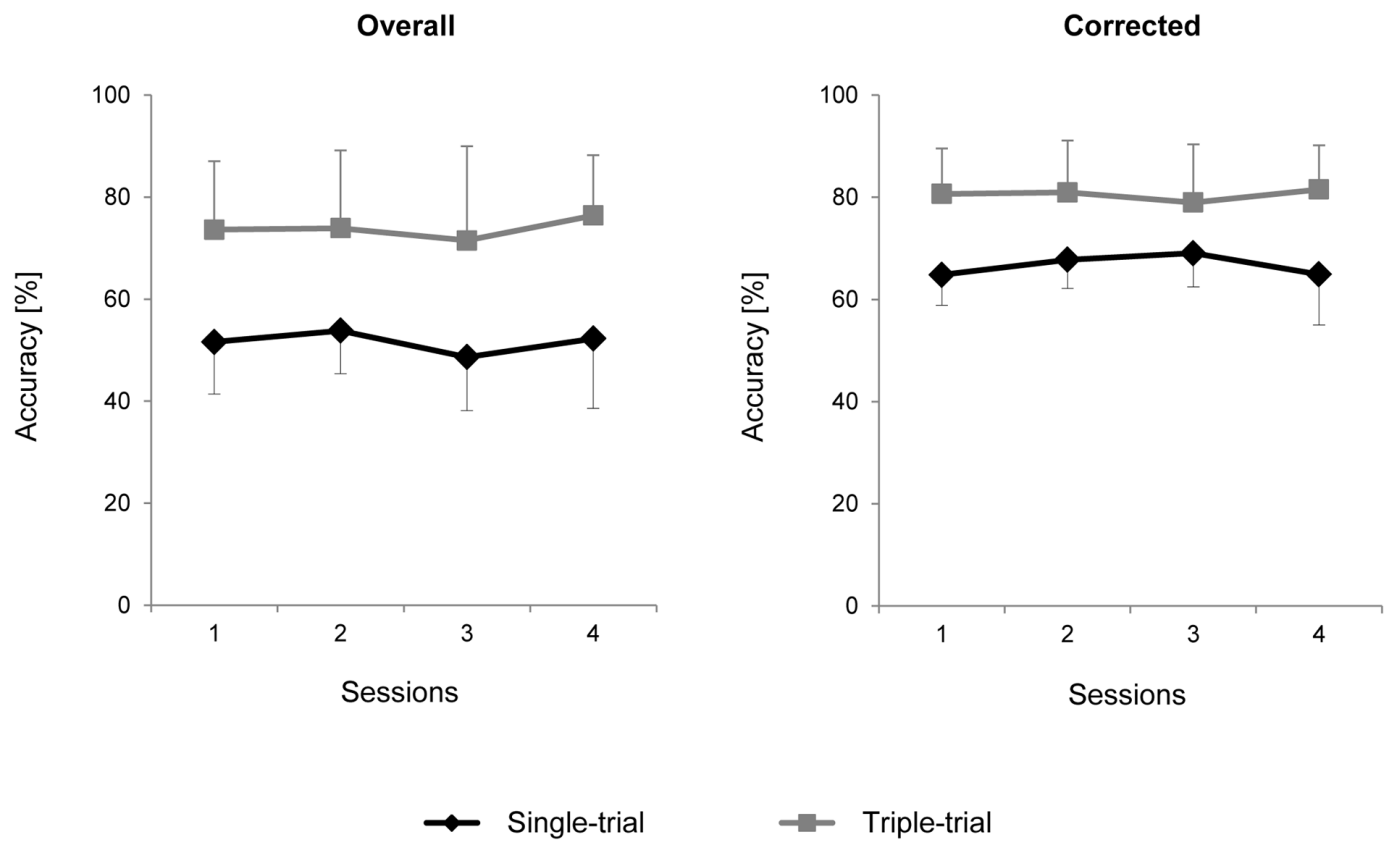
Online classification results for overall and corrected classification accuracy. Black diamonds, Single-trial group. Gray squares, Triple-trial group. Means ± standard deviations, in %.

Figure 3 shows group mean online accuracies per session and their standard deviations. In the ST group, the participants’ online overall accuracy per session in the main modes was in the range 31…65%, and in the TT group, the range was 39…97%. Thus, all of the subjects in all sessions performed better than at the random level, which was 11% (because one item should be chosen of nine items). Furthermore, corrected classification accuracy, i.e., accuracy computed without taking into account further attempts to select the target if the first attempt failed, was 53…74% in the ST group and 61…97% in the TT group (again, per participant and session). When individual accuracies were averaged over four sessions, the group *M*±*SD* for overall accuracy was 52±10% in the ST group and 74±13% in the TT group, and for corrected accuracy, 65±7% in the ST group and 81±9% in the TT group. Three-way MANOVA (Accuracy Type x Group x Session) revealed significant effects for Accuracy Type (Wilk’s λ = 0.12, *F*(1,10) = 76.0, *p* = 0.000006) and Group (*F*(1,10) = 11.1, *p* = 0.008) and their interaction (Wilk’s λ = 0.53, *F*(1,10) = 9.0, *p* = 0.013), whereas the Session effect (Wilk’s λ = 0.80, *F*(3,8) = 0.7, *p* = 0.59) and Session interactions with the other factors were not significant. 

To determine whether the four-session practice in Single-Trial compared with Triple-Trial mode leads to a different level of BCI control, a test with five stimulus sequences was run in the 2nd part of the 4th session. Both overall and corrected accuracy in this test was higher in the ST group than in the TT group (the corresponding medians were 91% and 75% for overall accuracy and 91% and 82% for corrected accuracy; *M*±*SD* for the overall accuracy was 85±16% for the ST group and 77±11% for the TT group and for the corrected accuracy, 88±12% and 83±08%, respectively). A two-way ANOVA (Accuracy Type x Group) again showed a significant effect for the accuracy index type (*F*(1,10) = 13.3, *p* = 0.005) but not for the factor of difference between the groups (*F*(1,10) = 0.84, *p* = 0.4) or its interaction with accuracy type (*F*(1,10) = 0.80, *p* = 0.4).

### Interest

**Figure 4:**
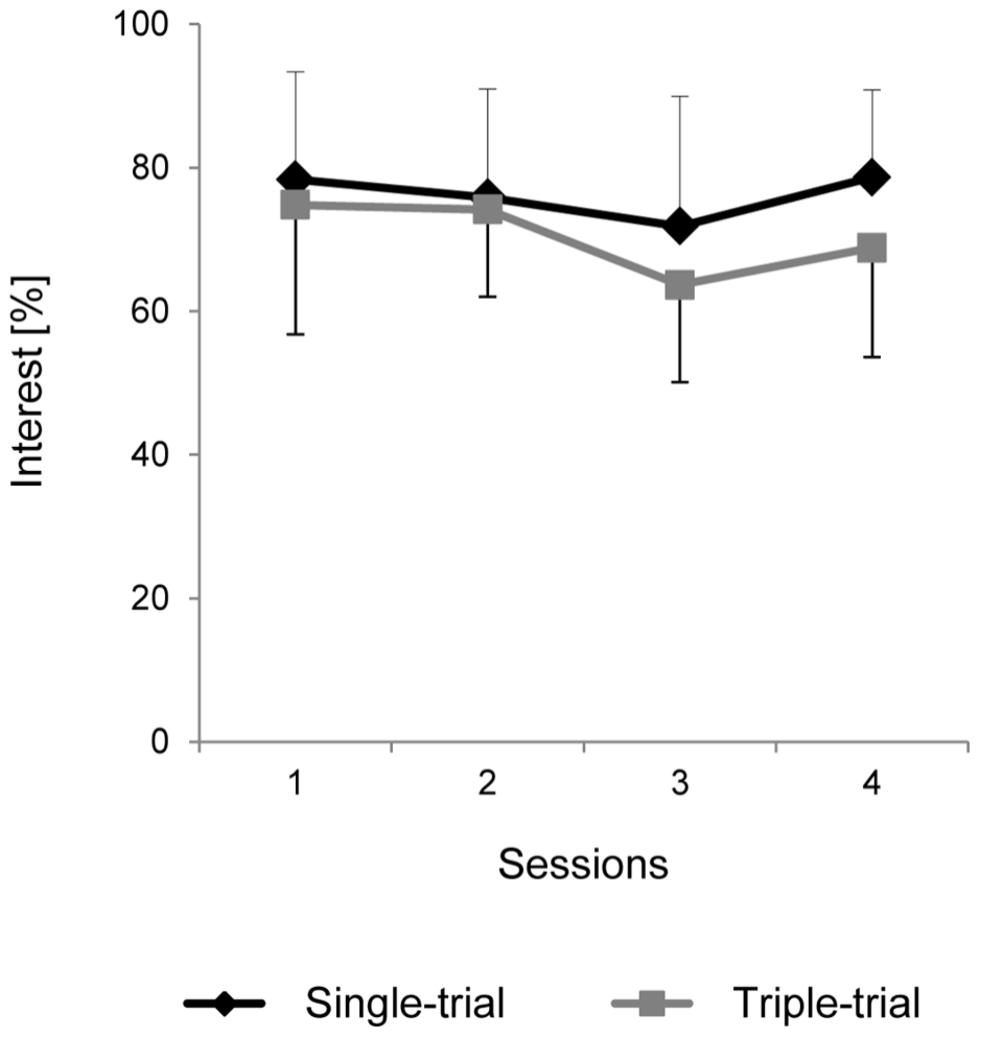
Interest in the task. Interest was self-estimated by the participants using a visual analog scale (VAS) at the end of each session. Black diamonds, Single-trial group. Gray squares, Triple-trial group. Means ± standard deviations, in % (corresponding to mm on a 100 mm-long scale).

Asked to report their interest in the task after each session, the participants in both groups indicated a high and generally stable level of interest (Figure 4). In the ST group, the interest self-estimate was 78±15% in the first session (here and later, *M*±*SD* is given) and 79±12% in the final session; the medians were 78.5% and 84%, respectively. In the TT group, the group-averaged interest values dropped from 75±18% in the first session to 69±15%, with medians of 76% and 70.5%, respectively. The lowest estimate of interest across all of the subjects and all sessions was 44%, i.e., only slightly lower than the midpoint (50%) of the interest scale. Two-way MANOVA showed no significant effects for the Session (Wilk’s λ = 0.79, *F*(3,8) = 0.71, *p* = 0.6) and Group factors (*F*(1,10) = 0.86, *p* = 0.4) or for their interaction (Wilk’s λ = 0.82, *F*(3,8) = 0.57, *p* = 0.6).

### ERPs

The number of epochs available for averaging significantly varied with the type of stimulus (the ratio of target to non-target stimuli was 1:8), group (a three times higher number of stimuli was presented to the TT than the ST participants) and game course (see “Methods” section). In addition, approximately half as much data were available in the 4th session than in other sessions, and minor variations were also added by rejection of varying numbers of epochs with artifacts. As a result, the number of available target epochs per session and participant ranged from 65 (participant 4 from the ST group, session 4) to 468 (participant 5 from the TT group, session 2). However, even the lowest numbers of available epochs appeared to be sufficient to obtain stable waveforms. We therefore decided to use all of the non-rejected epochs in each condition and group for averaging, although we considered the inequality of their amounts in discussing the results and drawing conclusions. For computing grand average waveforms and for the analysis of the P300 amplitude, two participants (#8 from the ST group and #12 from the TT group) were excluded from the analysis as having a too-small P300 component, which appeared to be nearly replaced by a positive wave peaking at approximately 200 ms. In addition, all of the data from participant 10 were not used for computing the Triple-Trial grand average, and his 1st-session data were treated as missed in the amplitude analysis because he had abnormal waveforms in the first session.

**Figure 5:**
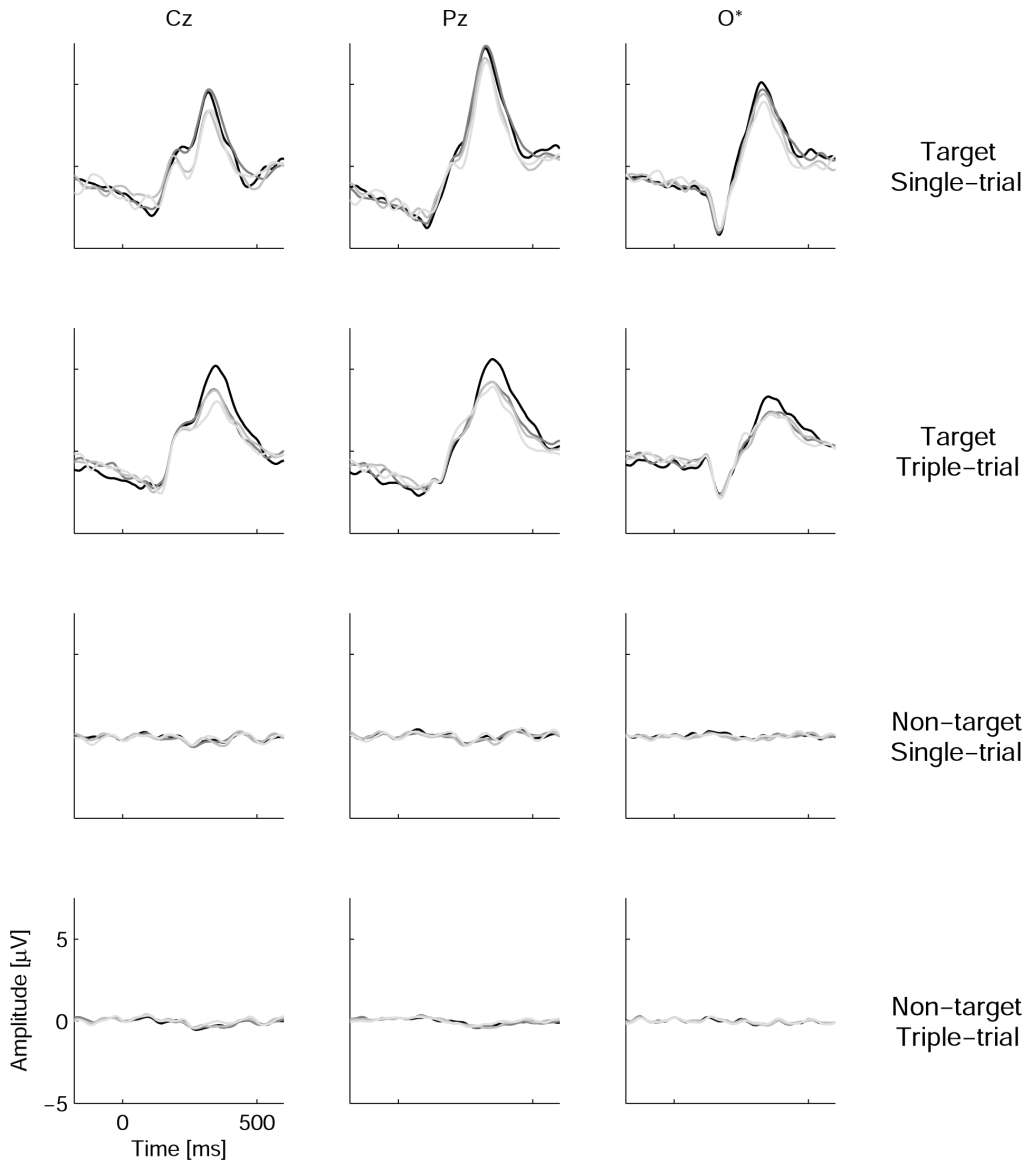
Grand average event-related potentials. Here, *n*=5 for each session in the Single-trial group (participant 8 excluded) and *n*=4 for each session in the Triple-trial group (participants 10 and 12 excluded). O* denotes an average of four occipital channels (PO7, PO8, O1 and O2). Zero time corresponds to the beginning of the stimulus. Waveforms are presented in black for the 1st session and in dark, medium and light gray for sessions 2, 3 and 4, respectively.

**Figure 6:**
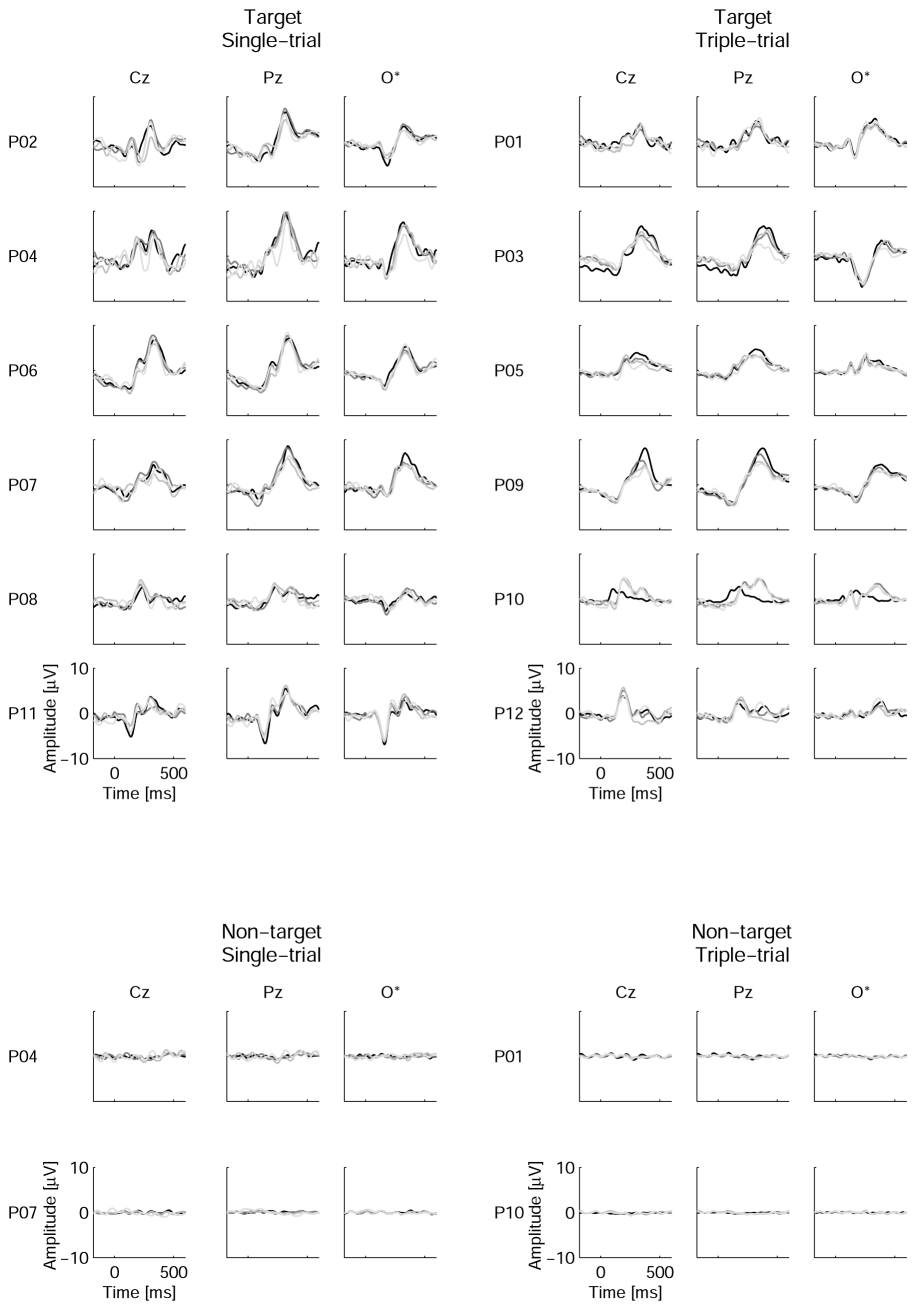
Individual averaged event-related potentials. For non-target ERPs, only data from representative participants are shown (P04 and P01 had relatively high-amplitude averaged responses to non-targets, whereas P07 and P10 represented those with especially low-amplitude non-target-related averages). O* denotes an average of four occipital channels (PO7, PO8, O1 and O2). Zero time corresponds to the beginning of the stimulus. Waveforms are presented in black for the 1st session and in dark, medium and light gray for sessions 2,3 and 4, respectively.

The grand average showed well-defined, high-amplitude waveforms for the EEG epochs related to the target stimuli and was very similar to a flat line in the case of non-target epochs (Figure 5). Individual ERPs (Figure 6) confirmed that in all of the participants, responses to targets had amplitudes of at least several microvolts, whereas responses to non-targets demonstrated only very low amplitude oscillations. 

**Figure 7:**
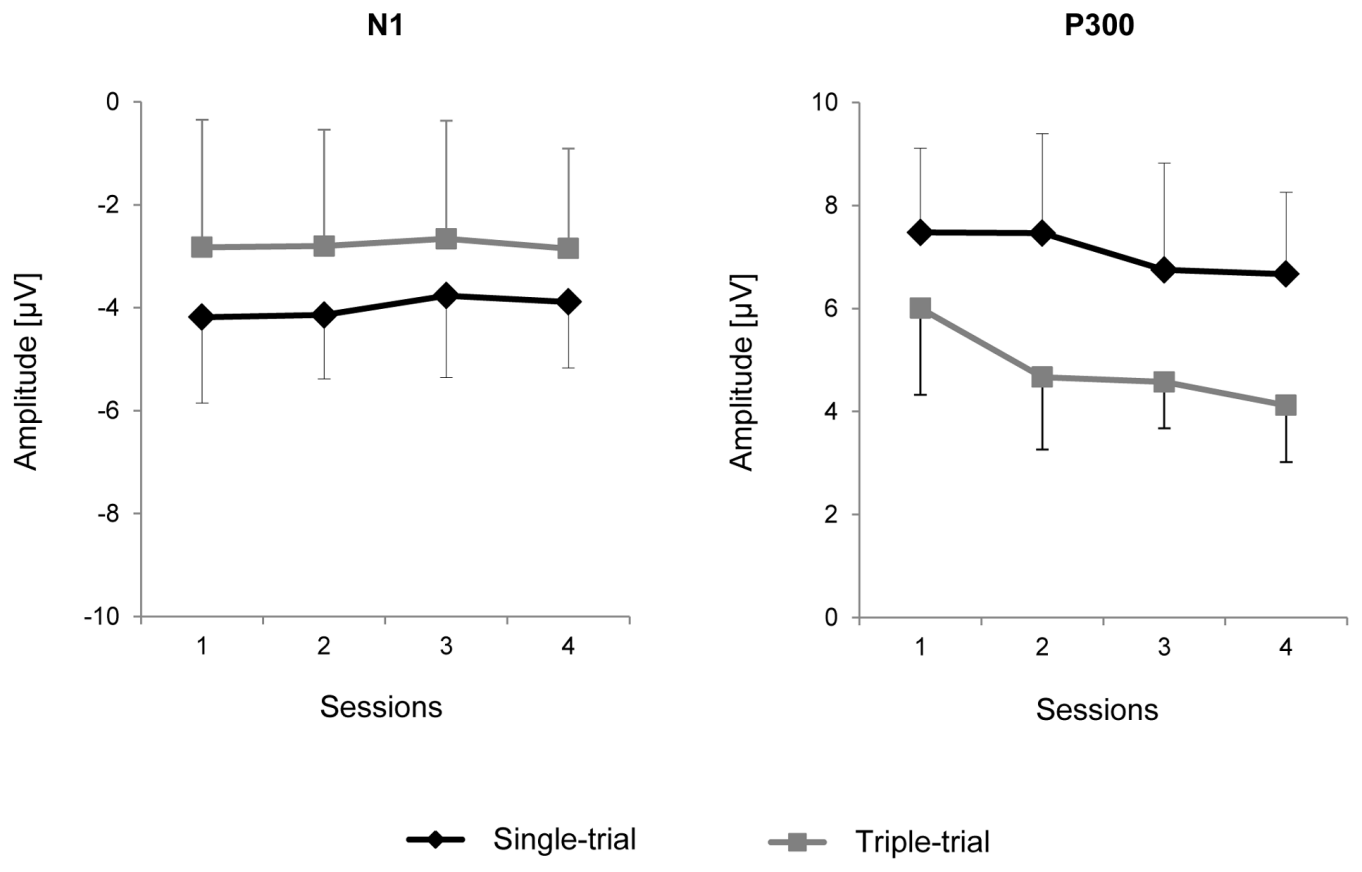
Amplitude of event-related potential peaks N1 and P300. Black diamonds, Single-trial group. Gray squares, Triple-trial group. Means ± standard deviations, in *µV*.

Group means and standard deviations for N1 and P300 amplitudes in response to target stimuli are shown in Figure 7. A two-way MANOVA (Group x Session) was applied separately to these components’ amplitude measurements. For N1, no significant effects were found (for Group, *F*(1,9) = 0.88, *p* = 0.4; for the repeated-measurement factor Session, Wilk’s λ = 0.76, *F*(3,7) = 0.73, *p* = 0.6; for the interaction between the two factors, Wilk’s λ = 0.83, *F*(3,7) = 0.48, *p* = 0.7). However, the group mean amplitude was higher in the ST group than in the TT group in all four of the sessions (Figure 7A), and it is possible that the difference did not reach significance due to small group sizes.

P300 amplitude data are shown in Figure 7B. In general, the ST group had a significantly higher P300 amplitude compared with the TT group (*F*(1,7) = 6.0, *p* = 0.04) and a marginally significant tendency toward a decrease in the P300 amplitude across sessions (Wilk’s λ = 0.25, *F*(3,5) = 4.9, *p* = 0.06). The interaction between Session and Group was not significant (Wilk’s λ = 0.33, *F*(3,5) = 3.4, *p* = 0.11). *M*±*SD* values were 7.5±1.6, 7.5±1.9, 6.7±2.1 and 6.7±1.6 µV in the ST group and 6.0±1.7, 4.7±1.4, 4.6±0.9 and 4.1±1.1 µV in the TT group in sessions 1-4, respectively. 

In addition to the N1 and P300 components, a positive component preceding the P300 peak was visible, mainly at Cz, in each participant’s ERPs. 

### Comparing Individual Dynamics

**Figure 8:**
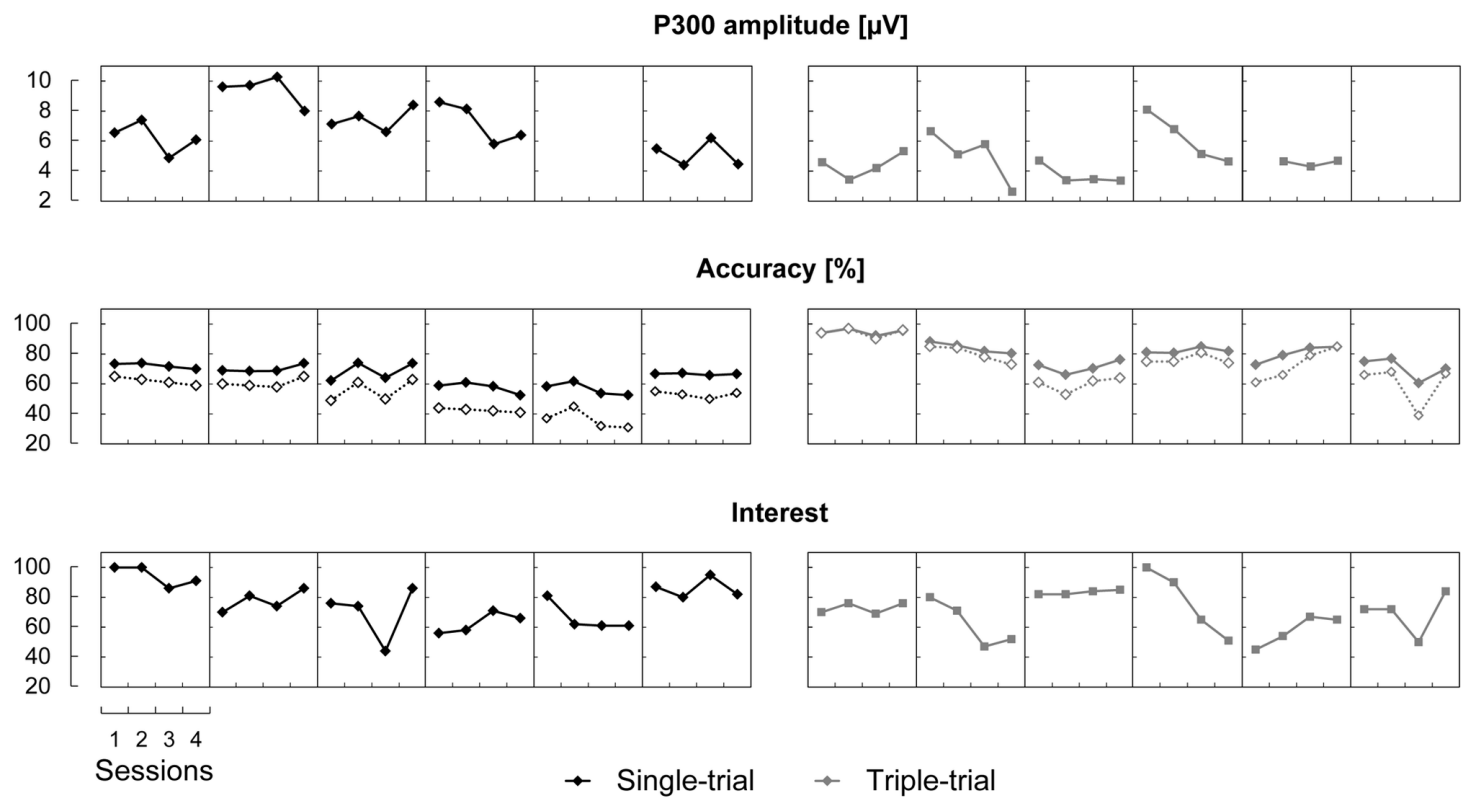
Dynamics of the individual index values across the four sessions. Each circle depicts the index value per session and participant. Lines connect the index values for each separate participant. For classification accuracy, overall accuracy data are shown by empty diamonds connected by dotted lines, and corrected accuracy data are shown by filled diamonds connected by solid lines. P300 amplitude data could not be measured for two participants or during one session for another participant.

Individual values of the P300 amplitude, of both accuracy indices and of interest are shown in Figure 8 for each of the four sessions. In addition to what was already shown by statistical analysis of the group data, the plot suggests that monotonous dynamics of accuracy and interest across sessions was typical in these data, although the direction of change varied across participants. The P300 amplitude, accuracy and interest appeared to be correlated across sessions. However, small numbers of sessions and small group sizes did not allow a detailed analysis of the dynamics of the variables and the relationships between them.

## Discussion

### Performance of the P300 BCI with moving stimulus positions

In the standard design of the P300 BCI, stimuli are presented at fixed positions. In this study, we showed, for the first time, that the P300 BCI can efficiently recognize a user’s intentions when stimuli are presented on separate moving objects, permanently changing their positions relative to each other.

In our earlier study [13], the ERP amplitude and classification accuracy remained stable, compared with a still condition, when the P300 BCI stimulus matrix moved at a speed of 5°/s or 10°/s, and the amplitude and accuracy decreased only at a higher speed (20°/s). However, in that study, the stimulus positions were fixed relative to each other. Therefore, non-target stimuli formed a spatially stable neighborhood near the target, so that the experiment’s participants always knew where non-target stimuli could be expected. This expectation was not the case in the current study, in which non-target flashes could appear in any direction and at a wide range of distances relative to the target. The non-target objects moved around the target and collided with each other and with the target, and the non-targets’ “distractive force” could be even further enhanced by their different colorful content. Frequent changes in the movement of the pursued target could produce frequent oculographic artifacts. In the Triple-Trial mode, several of these effects could be partly compensated for because the effects could not be frequently repeated in the same way in subsequent trials, but one could expect that accuracy in the Single-Trial mode would deteriorate very significantly. Experimental evidence was strongly needed to ensure that P300 BCI modification with stimuli presented on moving objects could maintain acceptable accuracy. 

The current study can be compared with the single-trial study by Finke et al [38], in which the task was also organized in the form of a BCI game. In both cases, stimuli were presented in a single-cell fashion, i.e., one item flashed at a time, without grouping into groups of simultaneously presented stimuli (e.g., rows/columns in the most typical form of the P300 Speller). The number of choices and target-to-target interval (which is critical for P300 amplitude [92]) were not significantly different in the two studies. However, the stimulus positions in the BCI design of Finke et al were fixed, whereas in our BCI, the stimuli moved permanently, at a speed of 5.4°/s. Despite this difference, the online accuracy achieved in the present study in single-trial mode (group mean of 65%) was approximately the same as in [38] (66%). In the present study, a degree of impact on accuracy could be made by self-initiation of the stimulation because this self-initiation could help the participants to be more prepared for the beginning of the stimulation. Furthermore, the highly varying fragments of color photographs used in the present study could possibly attract more attention than the non-varying stimuli in the study by Finke et al.. This factor could be especially serious for our corrected accuracy estimation because only first attempts with each new stimulus were taken into account. In addition, certain images used in the current study displayed baby animals, and it was recently shown that observing such images may improve the subsequent performance of tasks requiring focused attention [93]. However, variations in stimulus features could lead to greater brain response variability and therefore adversely influence the accuracy. One should also consider that we used a less advanced classifier compared with the classifier used by Finke et al.. Lastly, the time spent on classifier training was significantly lower in the present study: we used 1080 epochs collected in approximately 5 min (similar to [7]), whereas Finke et al [38] collected 6000 epochs, which should take at least 12 min at their stimulation rate. Taking all of these considerations together, we suppose that introducing movement into the P300 BCI design, almost certainly, did not lead to serious deterioration in BCI performance in our healthy users. 

Even if a proportion of the decrease in the BCI classification accuracy (e.g., due to a possibly lower N1 amplitude; see below) is caused by the movement of the stimulus positions, this phenomenon should not prevent such movement in applications in which the movement might be important. Examples of such applications, as we discussed in the Introduction, are games and, in certain cases, separately moving parts of robotic devices.

We did not include a control condition with stable stimulus positions in the present study because our main goal was only to answer the question of whether a certain type of BCI control is possible under moving conditions. The answer to this question was a decisive “yes”. The above comparison of classification accuracy between our study and other studies suggests that the movement of stimulus positions likely did not even lead to serious deterioration in performance. However, a comparison with the accuracy obtained under no-movement conditions would of course be needed to obtain a clearer answer regarding the possible effect of movement on P300 BCI accuracy.

### Target and non-target waveforms in the P300 BCI with moving stimulus positions

A comparison of the ERP obtained in the present study with published data can be made only in a limited way because, to our knowledge, ERPs have not been studied in detail in the case of single-cell (ungrouped) P300 BCI design. Guger et al [7] reported higher target P300 amplitude values in a single-cell protocol (8.8 µV) than those values obtained in the current study (6.7-7.5 µV in the single-trial mode and 4.1-6.0 µV in the triple-trial mode, depending on the session). The authors used 15 trials (15 flashes) per character, which could cause a degree of habituation (note the difference between the single-trial and the triple-trial P300 amplitudes in the present study) but much longer target-to-target intervals (because a selection was made from 36 possible choices, compared with nine in the present study). Taking all of these factors together, the difference between their and our P300 amplitudes does not appear prominent, despite the attentional and perceptional challenges raised by the movement of the stimulus positions. 

The N1 component was not analyzed in the single-cell P300 BCI. The absolute value of the N1 amplitude was similar to the P300 amplitude in row-column [13,29] and Hex-o-Spell [29,48] designs. In the current study, however, the N1 absolute amplitude was nearly two times lower than the P300 amplitude. In a previous study (Ganin I.P. (2010). Stability of the N1 component of brain potential in the P300-based brain-computer interface. Unpublished M.S. thesis. Lomonosov Moscow State University.), we found that the spatial distance between stimulus positions in the P300 BCI matrix and even removal of non-targets did not affect both the N1 and the P300 amplitudes in ERP-difference waveforms (the difference between responses to target and non-target stimuli), except in the case of a very short distance. Thus, variations in the distances between stimulus positions during motion were unlikely to directly cause changes in the ERP. Considering the dependence of the N1 component on gaze [29,55,56], one may hypothesize that the main cause of the possible effect of movement could be gaze tracking errors, such as insufficiently stable target fixation or occasional gaze slips from the target. 

One could suggest that false reactions to non-target stimuli could be more severe in the case of movement on separate trajectories, as attention and gaze might be captured by non-target objects when these objects approach the target object and collide with it. However, the amplitude of the responses to non-targets was very low (figures 5 and 6). A similar observation was reported for the “checkerboard” stimulation protocol [32], which is close to the single-cell design used in the present study in the sense that it avoids presentation of the non-targets at positions adjacent to the target. The non-target ERP amplitude in this protocol, which was under the same stimulus rate (8/s) as in the present study, was similar to what we observed but much lower than in the authors’ recordings using the standard row-column protocol. Townsend et al noted that “non-target items in scattered groups of items are less likely to attract attention than non-target items in entire rows or columns from the flanker effect (e.g., Sanders and Lamers, 2002), or from the Gestalt law of grouping (e.g., Prinzmetal, 1981)” ([32], p. 1118). Non-target ERPs in the single-cell design of [26] had high amplitudes, but this phenomenon may be due to large, closely positioned visual stimuli. In the present study, non-target flashing at positions close to the target was not excluded but was rare. It appears that the combination of single-cell design and moving stimulus positions in the present study could even more efficiently prevent Gestalt grouping.

### Possible benefits from stimulus position movement for BCI psychophysiological machinery

The important difference between the P300 BCI paradigm and the oddball paradigm remains when the stimulus objects move on different trajectories: the non-target positions are spatially distinct from the target position. Moreover, the non-target objects may not move in the same direction at the pursued target, which was the most typical situation in our experiment. Thus, only projections of target stimuli were expected to be fixed on the retina due to pursuit, whereas the non-targets moved on the retina. Note that pursuit is easily initiated and generally easily maintained in response to a smoothly moving target (see, e.g., [94]). 

This situation may have certain benefits for differentiating target and non-target stimuli using the ERP. More attention is allocated to pursued stimuli than to stimuli moving in the same direction but with different velocities [95]. When moving objects are not pursued, these objects are perceived less efficiently, and under certain conditions, prominent changes in their luminance and other characteristics can occur without the awareness of the observer [96]. Our observation of a very low non-target ERP amplitude may be partly linked to the effects of these types (in addition to the factors mentioned above). However, the conditions under which these effects appear were not yet studied in sufficient detail to allow predictions of whether the effects would be present in our design. In fact, when asked whether they perceived the target flashes as brighter than the non-target flashes, only half of the participants practicing in the Triple-Trial mode and none from the ST group responded positively. Additional studies are needed to clarify whether stimulus item movement can help P300 BCI users to perceive the targets better than the non-targets.

A study with non-flashing stimuli [66] demonstrated that attention during pursuit is focused in a 2° area centered on the target, which would also be beneficial for a BCI. However, in studies employing flashing moving stimuli [97,98], it was found that attention is biased toward an area in front of the pursued stimulus. It is unknown whether a non-target stimulus appearing in this area can trigger a stronger ERP.

Moving stimulus positions may have an additional benefit from a “screensaver” effect. During relatively long gazing at the target, which is required by the conventional P300 BCI paradigm, the image projection to the retina remains stable. Special mechanisms, such as microsaccades, should be activated under such conditions to prevent otherwise imminent retinal fatigue [99]. When stimulus positions move, non-target stimuli are excluded from causing such fatigue. Note that smooth pursuit is optimized for keeping the retinal image within the foveal area rather than for static fixation [57]. Thus, certain movement of the pursued object’s image on the retina is inevitable. Watching moving targets under certain conditions might therefore be even less difficult for the visual system than gazing at a stable P300 BCI stimulus matrix, at least in the users who are controlling their gaze. This argument for the use of movement is currently highly speculative and needs to be tested in more specifically oriented experimental studies. It is worth noting, however, that the same logic can be applied to BCIs based on the steady-state visual evoked potentials (SSVEPs), for which the problem of visual fatigue is serious [100]. Therefore, the use of stimulus position motion in the SSVEP BCI framework may be prospective.

### Stability of interest in the task

Because the P300 BCI worked well under the condition of stimulus position movement, we looked for an answer to an additional question: would the users of this BCI maintain their interest in the task after several sessions? The participants’ self-estimates showed that interest was high in all four of the sessions (Figure 4). This result is further supported by the fact that none of the participants refused to continue their participation up to the 4th session, despite the available option to cancel at any stage and despite the lack of monetary or other compensation for the time spent. 

The absence of a control group practicing with a standard P300 BCI (with stable stimulus positions) made it impossible to decide whether the high and stable interest was a result of adding movement to the interface or the other details of the interface design could be already sufficient. Moreover, the other elements of the experimental procedure could, in principle, bias the estimates (e.g., we cannot exclude that the participants reported high interest partly because they felt that this interest was what the experimenters wished to observe). Therefore, the high values obtained in this study, and even the fact of stability across sessions, should be treated as very preliminary results. 

However, even this preliminary evidence is important. In contrast to severely paralyzed patients, healthy participants in experiments typically do not feel a real need for the long-term use of BCI control. Although excitement can be observed when a healthy participant uses a BCI for the first time [78], he or she may consider stereotyped BCI tasks uninteresting within several sessions, and his or her attention and attention-related ERP components can be adversely affected. The stability of a healthy user’s interest in our new modification of the P300 BCI may create a basis for its use in multisession experiments with such participants. 

Interest estimations obtained across different sessions in the same participant can be studied in relation to classification accuracy and the amplitude of ERP components. The corresponding plot (Figure 8) suggests that these measures may be correlated. However, the number of sessions was too small for statistical analysis. In addition, we measured interest only at the end of each session, and it was unclear whether interest influenced the P300 amplitude and accuracy or accuracy affected interest. 

Kleih et al [78] did find a correlation between self-estimated motivation and the P300 amplitude in P300 BCI users by between-subject analysis. However, intra-individual analysis appears to be less vulnerable to variations between participants in irrelevant factors, such as different understanding of the scales and different “internal scales” for psychological variables. It will be important to use intra-individual analysis of correlations between interest, motivation and other subjectively assessed factors in experiments with sufficiently high numbers of sessions to reveal the psychological determinants of P300 BCI performance.

Importantly, the high level of interest in the present study was maintained under conditions in which the number of errors was high due to the absence of averaging. Involvement in a game could cause an attitude toward errors that is substantially different from what is typical for non-game-like tasks; in a game, it is natural to consider error-forcing conditions as a challenge [68]. Finke et al [38], in a single-session study, already attempted to intensify practice by combining a single-trial P300 BCI and game design. However, making the task more engaging by adding movement could, in principle, ensure better training conditions.

For issues that can be relevant to further improvement in a user’s interest and involvement in the task using game-like settings, the reader may refer to the discussion on the use of games to improve participants’ attitudes toward psychological experiment requirements in [101]. The reader may also refer to a review of P300 BCI features preventing the use of the P300 BCI in games and the ways to overcome these issues in [8].

### No practice effects in four sessions

Due to the relatively high interest in the task revealed by the participants, we would expect that the participants were sufficiently involved in their task. We therefore hypothesized that the positive effects of practicing with the P300 BCI in Single-Trial and/or Triple-Trial modes could lead to significantly increased performance within several sessions. Such an effect on performance was not observed. Moreover, we found a marginally significant P300 amplitude decrease across sessions, although it appears likely that this decrease did not reach the significance threshold only due to the small group sizes. 

A latent effect of practice on P300 BCI control skills could be revealed by a test with a more standard number of stimulus repetitions (five) run in the second half of the final session, in the form of higher performance of the group that practiced in the mode enabling more efficient training. Due to the absence of EEG response averaging, the Single-Trial mode was supposed to be more likely to enable efficient training. The group practicing in Single-Trial mode indeed showed better performance (88%, in contrast to 83% for the TT group), but the difference was not significant. 

Arico et al [102] reported an increase in the *R* ^*2*^ index measuring the difference between target and non-target brain responses after three sessions of P300 BCI practice with eight repetitions of stimuli. However, the sessions were short (three runs and six character selections per run), and it is possible that the changes that the authors observed were specific to the beginning of the participant’s familiarization with the BCI. Moreover, the researchers’ channel plot shows that the effect was not observed in the channels in which the P300 amplitude is high (Fz, Cz and Pz) but was specific for occipital channels and thus specific for the earlier negative component, which is gaze dependent [29,55]. Thus, the effect could be caused by such factors as gaze stabilization and not by feedback-based enhancement of the activity of ERP “generators” in the brain.

The absence of accuracy improvement based on presumably improved feedback in the present study may be related to the excessive difficulty of the participants’ task. As we discussed above, various effects of movement and specific features of the stimuli (e.g., different bright colors) could increase attention distraction. Several “balls” appeared to be overly difficult targets, as indicated by a positive difference between the corrected and the overall classification accuracies (i.e., the participants tended to fail again after not correctly selecting the target in the first attempt). Note that the “noise” produced by “machine errors” could decrease the quality of feedback (indeed, the participants practicing in the Single-Trial mode often reported that they did not feel good control of the BCI). All of these factors could create conditions that were not optimal for conditioning-based or other practice effects. Note that testing BCI practice effects was only an additional objective of the current study and was performed in a preliminary way. The only detail of the study design specifically introduced to improve conditions for practice was the use of a mouse for stimulation initiation when the participants presumably found themselves more prepared for the task.

It is possible that four sessions were simply not enough for efficient learning. However, it is evident that before undertaking a study with a higher number of sessions, efforts should be made to improve feedback by minimizing the rate of errors unrelated to intrinsic attention fluctuations. In particular, it might be important [1] to use more equalized stimuli and to exclude pictures for which an especially high error rate was observed [2], to decrease the distracting influence of the moving object collisions and their close convergence (e.g., by setting a distance threshold) and (3) to choose computational algorithms and their parameters more carefully (e.g., higher accuracy may be achieved using more advanced classifiers, and faster feedback may be enabled by using FIR filters and shorter stimulus related epochs, for example). Gradual feedback [38] should also be considered, although with caution, because the feedback signal in this case is very noisy, and the noise variations in the signal may act as an additional distractor. 

### A positive wave preceding the P300

We observed a positive peak at Cz preceding the P300 wave in all the participants. In most participants, the peak was visible in all four of the sessions. This little studied component is often observed in P300 BCI research, occasionally apparently replacing the “standard” P300 (e.g., in [32]; see also [29]). Detailed analysis of this component was not possible using our data due to high contamination by the overlapping P300 (the low number of channels in our EEG recordings did not allow the correct use of demixing techniques, such as ICA or PCA). However, it is worth noting that we observed the peak even in the Single-Trial mode, so this peak could not be related, e.g., to a specific range of target-to-target intervals. 

### Issues related to the limitations set by paralysis

The potential users of the P300 BCI with moving stimulus positions will likely be people who have at least a certain degree of motor control (most of the users of assistive robotics) or have full motor control (most of the users of BCI games). However, BCIs are primarily designed to assist the most severely paralyzed people. These people could also benefit from the use of assistive robotics and even from playing BCI games for entertainment or for training in BCI control skills. However, these individuals cannot use a mouse to start the stimulation and cannot even efficiently use eye movements to pursue the target. The initiation of the stimulation using a mouse appears not to be a critical part of our BCI design and might easily be replaced by, e.g., a switch based on a motor imagery BCI or the use of an “asynchronous” P300 BCI approach [90,91]. It remains far less clear whether a patient with impaired gaze control could use a BCI when the target position is moving. This use does not, however, seem impossible because even multiple moving objects can be simultaneously tracked without eye movements, using covert attention only [59]. Processing of non-pursued stimuli is selectively enhanced if the observer is aware of the direction of motion [103,104], and it is possible that this effect can be exploited in the BCI design. In this case, it is likely that the number of moving objects should be lower than in the case of a P300 BCI with pursued moving objects, but a selection can even be made from many options using two or more steps, as in the “region-based” [49] or “Hex-o-Spell” [48] paradigms.

## Conclusion

This study investigated whether P300 BCI stimuli can be presented on moving objects without a dramatic loss of classification accuracy. Participants successfully operated a game-like interface despite the attentional and perceptional challenges raised by the movement of the stimulus positions. Moreover, the participants from both groups, practicing in either Triple-Trial (*n*=6) or Single-Trial (*n*=6) mode, maintained interest in their task across four sessions run on different days. The proposed BCI stimulus design, therefore, can be considered as a prospective basis for BCI games and might become a useful model for studying the effects of long-term BCI use in healthy people who are not motivated to use a BCI for communication or for control of robotics. The results also suggest that different stimulus items in the P300 BCI can be placed on separately moving parts of robotic devices.
